# Tailoring the stress response of human skin cells by substantially limiting the nuclear localization of angiogenin

**DOI:** 10.1016/j.heliyon.2024.e24556

**Published:** 2024-01-21

**Authors:** Rosanna Culurciello, Ilaria Di Nardo, Andrea Bosso, Francesca Tortora, Romualdo Troisi, Filomena Sica, Angela Arciello, Eugenio Notomista, Elio Pizzo

**Affiliations:** a Department of Biology, University of Naples Federico II, 80126, Naples, Italy; b Department of Chemical Sciences, University of Naples Federico II, 80126, Naples, Italy; c Institute of Biostructures and Bioimaging, CNR, 80131, Naples, Italy; d Centro Servizi Metrologici e Tecnologici Avanzati (CeSMA), University of Naples Federico II, 80126, Naples, Italy

**Keywords:** Stress induced RNases, Stress granules, Cell homeostasis

## Abstract

Human angiogenin (hANG) is the most studied stress-induced ribonuclease (RNase). In physiological conditions it performs its main functions in nucleoli, promoting cell proliferation by rDNA transcription, whereas it is strongly limited by its inhibitor (RNH1) throughout the rest of the cell. In stressed cells hANG dissociates from RNH1 and thickens in the cytoplasm where it manages the translational arrest and the recruitment of stress granules, thanks to its propensity to cleave tRNAs and to induce the release of active halves. Since it exists a clear connection between hANG roles and its intracellular routing, starting from our recent findings on heterologous ANG (ANG) properties in human keratinocytes (HaCaT cells), here we designed a variant unable to translocate into the nucleus with the aim of thoroughly verifying its potentialities under stress. This variant, widely characterized for its structural features and biological attitudes, shows more pronounced aid properties than unmodified protein. The collected evidence thus fully prove that ANG stress-induced skills in assisting cellular homeostasis are strictly due to its cytosolic localization. This study opens an interesting scenario for future studies regarding both the strengthening of skin defences and in understanding the mechanism of action of these special enzymes potentially suitable for any cell type.

## 1 Introduction

All living organisms must effectively counteract environmental stressors, exposure to toxins and mechanical damage [1,2]. The ability to rapidly respond to perturbations in the cellular environment is essential for survival and a general translational attenuation represents one of the key strategies that the cell implements to deal with stress [2]. In eukaryotic cells the arrest of protein synthesis is generally mediated by the inhibition of the mTOR kinase that leads to a reduced phosphorylation of 4 EBP and a resulting inhibition of the interaction between translation initiation factors eIF4E and eIF4G [3]. Concomitant phosphorylation of eIF2a by stress induced kinases (PKR, GCN2, PERK or HRI) triggers the inhibition of eIF2B resulting in a reduced cellular pool of active eIF2–GTP and hence a diminished rate of recognition of the initiation codon [4,5]. All these fast stress-response steps require the involvement of specific components of the translation machine and induce cells to reshape proteome, redirecting mRNAs encoding non-essential ‘housekeeping’ proteins from polysomes to transient storage aggregates mRNAs, namely stress granules (SGs), and simultaneously allowing the selective translation of mRNAs encoding proteins directly involved in rescue operations [[6], [7], [8]]. In addition to stress-induced protein synthesis inhibition, notable is also the targeting of other RNA components of the translational machinery, in particular transfer RNAs (tRNAs) [9], abundant RNAs in the cell with a significant degree of chemical modifications in their composition. tRNA molecules provide a reservoir of RNA species whose function extends beyond translation, indeed, as widely reported by increasingly findings, even tRNA fragments (tRFs) can affect various cellular processes [10,11] and are involved in several human diseases [12,13]. Among these tRFs, a prominent role in cellular stress response, increasingly documented, is undoubtedly covered by angiogenin-cleaved tRNA halves (tiRNAs), some of which [14,15] are able to directly inhibit protein synthesis by displacing the translation initiation factor eIF4G from mRNA on ribosomes [16,17]. These active small noncoding RNAs are also involved in the regulation of mRNA stability, interaction with cytochrome C to modulate apoptosis and the SGs assembly [18]. The release of tiRNAs is mediated by angiogenin (ANG), a member of the Vertebrate RNase Superfamily [19,20], widely expressed in most tissues and associated to angiogenesis, hematopoiesis, oncogenesis, neurodegenerative diseases [21,22], inflammation, and immunity [23,24]. ANG is actively secreted and captured by cells both in an autocrine and in a paracrine way [25]. Beyond that, properties and subcellular localization of ANG are closely dependent on the growth status of the cells and by the interaction with RNH1 (RNase inhibitor 1) for which ANG has a very strong affinity (Kd ∼1 fM) [26,27]. When cells are under growth conditions, nuclear ANG is completely inhibited by RNH1 whereas nucleolar ANG is fully active to stimulate rRNA transcription [28]. Conversely, if cells are subjected to a stress, the majority of ANG moves to the cytoplasm and is activated by dissociation from RNH1 to cleave tRNAs and release tiRNAs, actively involved in the cellular response to stress [15,29]. Interestingly, previous studies reported that also recombinant ANG, when added directly to culture media of multiple cell lines, is rapidly internalized and cleaves mature cytoplasmic tRNAs within anticodon loops [16,30,31]. This emphasizes even more the meaningfulness of dynamic subcellular localization of ANG in the cell and above all how cytoplasmic localization is the essential requirement for handling altered cellular homeostasis. In this regard, it is worth noting our recent contribution to the study of the stress-induced role of ANG [17] where we highlighted the ability of human keratinocytes to intercept recombinant ANG from the culture medium and the benefits that this uptake guaranteed to the cells subjected to oxidative stress. These indications are in line with other reports that support the idea concerning the key role of ANG in aiding the cell, namely its ability to flow into the cytoplasm when cellular homeostasis is compromised and to ensure the release of tiRNAs [32,33]. Based on these considerations, in this report we performed an extensive functional characterization of an ANG variant in which the nuclear localization sequence (NLS), composed by residues ^31^R-^32^R-^33^R-^34^G-^35^L [34], was drastically altered. The goal behind this strategy is to draw a solid connection between the cytoplasmic localization of ANG in its active form and the ability of the protein to contribute effectively to the cell stress response. To do this, on the wake of our previous paper [17], we chose human keratinocytes as model cell line and designed a variant of ANG in which the substitution of two arginine residues (R_31_/R_32_) with two glutamine residues resulted in an almost total suppression of the net positive charge of the NLS. This double variant, to date never studied and named R31Q/R32Q, was produced in recombinant form and extensively studied for its rescue properties in stressed HaCaT cells in comparison with the properties previously reported for unmodified recombinant ANG.

## 2 Materials and Methods

### 2.1 Heterologous proteins production and purification

Recombinant production of human angiogenin (ANG) and its variant (R31Q/R32Q), both equipped with a C-terminus 6x-His tag, was obtained by using an expression and purification protocol already described for other RNases belonging to the same Superfamily [35]. Briefly, the expression plasmids pET-22b (+) encoding ANG or R31Q/R32Q were used to transform competent *E. coli* strain BL21 (DE3) (ThermoFisher Scientific, Waltham, MA, USA). Cells were grown at 37 °C to an A_600nm_ = 0.6, then induced with 0.4 mM IPTG (isopropyl-1-thio-d-galactopyranoside) and grown overnight (ON). Inclusion bodies were obtained after sonication and centrifugation, and recombinantly expressed proteins were then purified by RP-HPLC [35,36].

### 2.2 Circular dichroism analyses

Circular dichroism (CD) was carried out at 20 °C using a Jasco J-1500 spectropolarimeter and a cell with an optical path length of 0.1 cm. Spectra were registered with 50 nm min^−1^ scanning speed, 2 s D.I.T., 1 nm data pitch, and 2.0 nm bandwidth and averaging three scans. Thermal unfolding curves were obtained by following the CD signal at 220 nm, in the 20–85 °C range and at heating rate of 30 °C h^−1^. The denaturation temperatures (T_d_) were determined through analysis of the first derivative of the melting profiles. All CD experiments were performed with a protein concentration of 0.1 mg ml^−1^ in 20 mM MES/NaOH pH 6.0 and 100 mM NaCl.

### 2.3 Ribonucleolytic activity assays

The ribonucleolytic assays were performed incubating 50 μM of yeast tRNA (Sigma-Merck, Milan, Italy) with 2 μM of ANG or R31Q/R32Q. The reactions were performed in 25 mM KH_2_PO_4_, 1 mM MgCl_2_ and 50 mM KCl, incubated at 37 °C for 30 min and then blocked with 8 M Urea at 95 °C for 5 min. The electrophoretic separation was obtained in TBE 1X and RNA were then stained in Toluidine Blue 0.1 %.

### 2.4 Cell culture and treatments

HaCaT cells were cultured in Dulbecco's modified Eagle medium (DMEM) supplemented with 10 % fetal bovine serum (FBS), 1 mM l-glutamine, 1 % v/v penicillin/streptomycin solution (100 Unit/ml) and grown at 37 °C in 5 % CO_2_.

Oxidative stress was induced by incubating cells with 500 μM of Sodium Arsenite (SA) (Merck KGaA, Darmstadt, Germany) for 1 h at 37 °C.

Recombinant proteins were administrated for the biocompatibility assay at increasing concentrations for 24 h. For the following experiments, pre-treatments were carried out by using 2 μM ANG or R31Q/R32Q for 1 h.

Cell rescue was evaluated after different times, depending on the assay, by replacing the SA-containing medium with fresh medium.

### 2.5 Cell viability assays

For the biocompatibility evaluation of ANG or R31Q/R32Q, 5 × 10^3^ HaCat cells were seeded in 96-well plate and, after 24 h, treated with increasing amounts of recombinant proteins.

Cells viability was then assessed by MTT method [37], and analyzed at 570 nm by means of a Multi-Mode Microplate Reader (Synergy™ H4). Cell survival was expressed as the mean of the percentage values compared to control untreated cells.

### 2.6 Western blot analyses

Proteins from NE-PER™ nuclear and cytoplasmic extract reagent kit (ThermoFisher Scientific, Waltham, MA) were quantified by Bradford method (Sigma-Merck, Milan, Italy).

A total of 30 μg of nuclear and cytosolic protein fractions were separated by SDS-PAGE, and then electro-transferred to Immobilon-PVDF membranes (Sigma-Merck, Milan, Italy). After blocking in 5 % bovine serum albumin (BSA), PVDF membranes were incubated ON in primary antibodies: Rabbit β-actin polyclonal antibody (Sigma-Merck, Milan, Italy), mouse 6x-His Tag mAb (ThermoFisher Scientific, Waltham, MA) and rabbit PARP antibody (Cell Signaling Technology, Danvers, MA, USA, 1:1000). After washing, membranes were incubated with horseradish peroxidase (HRP) conjugated to goat anti-mouse or anti-rabbit IgG (1:5000) (ImmunoReagents Inc., Microtech SRL, Naples, Italy). Membranes were then incubated with ECL (Enhanced chemiluminescence substrate) and recorded by ChemiDoc Imaging System (Bio-Rad Laboratories S. r.l., Segrate (MI), Italy). Results were then subjected to densitometric analyses performed with the software ImageJ.

### 2.7 DCFH-DA assay

The ROS quantification assay was carried out by using the DCFH-DA (2′,7′-Dichlorofluorescin diacetate) [38].

2 × 10^4^ HaCat cells were seeded into 96-well plate and incubated at 37 °C in a 5 % CO_2_ atmosphere ON. Then, cells were washed in PBS 1X, opportunely treated, and incubated with 20 μM DCFH-DA, at 37 °C for 40 min. After the incubation time cell fluorescence from each well was measured and recorded in the excitation/emission wavelengths of 485–532 nm, by means a Multi-Mode Microplate Reader (Synergy™ H4).

### 2.8 Lipid peroxidation analysis

Lipid peroxidation products [thiobarbituric acid reactive substances (TBARS) also known as malondialdehyde-equivalents (MDA-equivalents)] from HaCaT cells were measured by the thiobarbituric acid colorimetric assay, as previously reported [39,40].

Briefly, HaCaT cells were seeded in 6-well plates at density of 2.5 × 10^5^ cells/well. Cells were then opportunely treated, washed with PBS, counted and centrifuged at 500 rpm for 5 min. After removal of supernatant, 0.5 ml of ice-cold 40 % trichloroacetic acid (TCA) and 0.5 ml of 0.67 % of aqueous thiobarbituric acid were added to the pellet. The mixtures were heated at 90 °C for 15 min, then cooled in ice for 10 min, and centrifuged at 800×*g* for 10 min. The supernatant fractions were collected, and lipid peroxidation estimated spectrophotometrically at 530 nm. The amount of TBARS formed was calculated using a molar extinction coefficient of 1.56 × 10^5^/mol/cm and expressed as nmol TBARS/10^6^ cells.

### 2.9 Real-Time quantitative PCR (RT-qPCR)

Gene expression in HaCaT cells was evaluated by RT-qPCR. In brief, 3 × 10^5^ cells were plated and after 24 h exposed to treatments (see paragraph 2.4. and 3.3.). Total RNA was then isolated using TRIzol™ Reagent (ThermoFisher Scientific, Waltham, MA, USA) according to the manufacturer's instructions and 1000 ng of RNA were used for cDNA synthesis using SuperScript™ IV VILO™ Master Mix (ThermoFisher Scientific, Waltham, MA, USA) following the manufacturer's protocol. RT-qPCR was performed by using SYBR GREEN PCR MASTER MIX (ThermoFisher Scientific, Waltham, MA, USA) in the StepOnePlus™ Real-Time PCR System (ThermoFisher Scientific, Waltham, MA, USA). The expression of each gene detected (Table 1) was normalized with respect to GAPDH gene (housekeeping gene control).

**Table 1:** Primer sequences associated to selected genes.

Genes	Forward (5′–3′)	Reverse (5′–3′)
GAPDH	CACCACACTGAATCTCCCCT	TGGTTGAGCACAGGGTACTT
HSPA6	TGCAAGAGGAAAGCCTTAGGGACA	TTTGCTCCAGCTCCCTCTTCTGAT
NQO1	TATCCTTCCGAGTCATCTCTAGCA	TCTGCAGCTTCCAGCTTCTTG
GCLC	TATCCTTCCGAGTCATCTCTAGCA	TCTGCAGCTTCCAGCTTCTTG
SOD	GAAGGTGTGGGGAAGCATTAAAG	GAAGGTGTGGGGAAGCATTAAAG
CAT	CGGAGATTCAACACTGCCAATG	TTCTTGACCGCTTTCTTCTGGA

### 2.10 Immunofluorescence

Immunofluorescence analyses were performed on 2,5 × 10^4^ HaCaT cells seeded on glass coverslips in 24-well plates and cultured for 24 h. After treatments (see paragraph 2.4. and 3.3.), cells were fixed in 4 % paraformaldehyde (PFA), permeabilized in 0.1 % Triton X-100 and blocked with 1 % BSA. Then cells were incubated ON with primary antibodies, suitably diluted as indicated by the manufacturer, in 1 % BSA. Primary antibodies used were: mouse 6x-His Tag mAb (Thermo Fisher Scientific, Waltham, MA), rabbit PABPC1 pAb (Sigma-Merck, Milan, Italy), mouse PABP mAb (Sigma-Merck, Milan, Italy). After washing in PBS, the coverslips were incubated with secondary antibodies Alexa 488 conjugated goat anti-rabbit (Thermo Fisher Scientific, Waltham, MA) or Cy5 conjugated goat anti-mouse F (ab’)2 (1:500 dilution) (Jackson Immuno Research Europe Ltd, Cambridgeshire, UK) for 1 h. Nuclei were stained with DAPI (Molecular Probes, Thermo Fisher Scientific, Waltham, MA) and then washed. Coverslips were finally mounted in Mowiol® 4–88 and analyzed using a Zeiss Confocal Microscope LSM 700 at 63× magnification.

Fluorescence intensity analysis was then obtained by using ImageJ *via* the formula reported:

CTCF = Integrated Density – (Area of selected cell × Mean fluorescence of background readings) as described by McCloy et al. [41]. The fluorescence intensity of each cell was calculated using Excel (Microsoft Office). For each image, three background areas were used to normalize against autofluorescence [42].

### 2.11 Statistical analyses

Statistical analyses were carried out by using GraphPad Prism. Values are reported as the means ± SEM of biological replicates (*p < 0.05, **p < 0.01, ***p < 0.001 or ****p < 0.0001) compared to the respective controls (one-way ANOVA, followed by Bonferroni's post-test).

## 3 Results and discussion

### 3.1 Design, production and ribonucleolytic properties of R31Q/R32Q

**Fig. 1:**
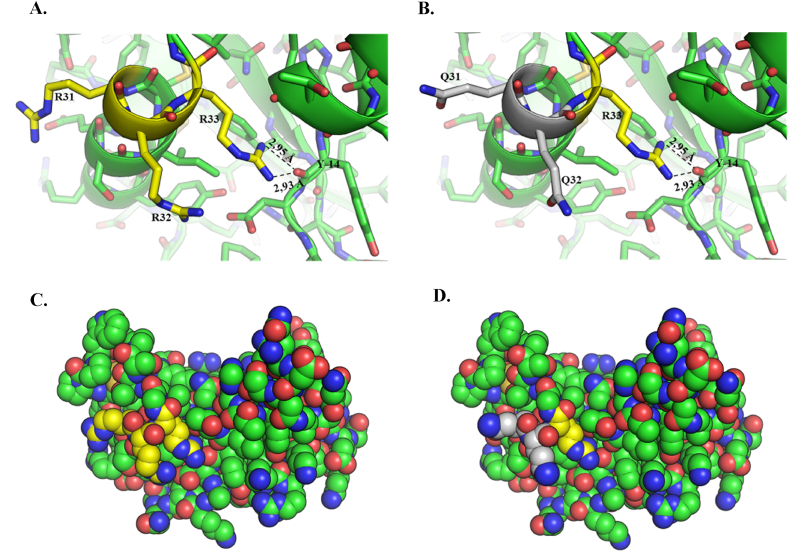
Structural overview highlighting the location of angiogenin (ANG – panel A) arginine residues, R31 and R32, replaced in the variant (panel B) by glutamine residues Q31 and Q32. In both panels it is also highlighted a tyrosine residue (Y14) involved in H bonds (dashed lines) with the amino group of the third arginine residue of parent protein NLS region (R33), conserved also in the variant. In panels C and D, the atomic surfaces of ANG and its variant are shown, respectively. In green carbon atoms (C), in blue nitrogen atoms (N) and in red oxygen atoms (O). In yellow the amino acids involved in mutations are highlighted. (For interpretation of the references to color in this figure legend, the reader is referred to the Web version of this article.)

As previously mentioned, ANG exhibits a dynamic subcellular localization strictly influenced by cell growth conditions. Here, we aimed to rationally design and express in heterologous form a variant of human ANG in which its nuclear/nucleolar localization sequence was severely compromised to induce a predominant cytosolic localization of the protein. As shown in the structural overview reported in Fig. 1, we replaced R31 and R32 of parent protein (Fig. 1 panel A and C) with two glutamine residues (Fig. 1 panel B). The main aim of this strategy was to drastically alter the NLS region by almost completely reversing its net positive charge and concomitantly preserving R33 to maintain contacts, by H bonds, with the main chain of Y14. In this way, the two replaced residues significantly spoil NLS region without theoretically altering the folding and the solvent exposure of the structural context adjacent to it (Fig. 1 panels B and D).

**Fig. 2:**
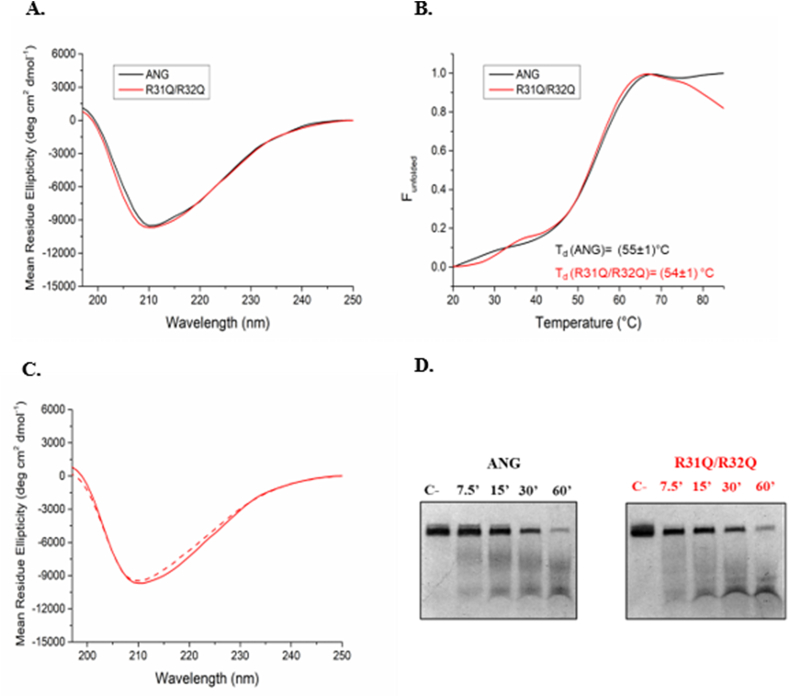
A. CD spectra of R31Q/R32Q and ANG 0.1 mg mL^−1^ recorded at 20 °C in 20 mM MES/NaOH pH 6.0 and 100 mM NaCl; B. thermal denaturation profiles and denaturation temperatures of R31Q/R32Q and ANG; C. superimposition of the CD spectra of R31Q/R32Q registered at 20 °C before heating (solid line) and after cooling (dashed line); D. UREA-PAGE analysis of the ribonucleolytic activity of R31Q/R32Q and ANG on yeast tRNA (C-: negative control represented by a tRNA mixture with a molecular size ranging between 70 and 90 nucleotides; 7.5′-15′-30′-60’: minutes of tRNAs incubation with ANG or R31Q/R32Q).

The designed variant, henceforth named R31Q/R32Q, was obtained by protein engineering, recombinantly produced, purified to homogeneity (see Materials and Methods section), and subsequently investigated in solution by CD spectroscopy where the inspection of the far-UV CD spectrum (Fig. 2 panel A) indicated that it retained the secondary structure of parent protein. Thermal unfolding curves were obtained in the temperature range 20–85 °C by following the CD signal at 220 nm (Fig. 2 panel B). CD spectra of R31Q/R32Q, recorded at 20 °C before heating and after cooling, indicated a reversible thermal unfolding (Fig. 2 panel C), as observed for the unmodified protein [18]. The denaturation temperature is comparable with that of ANG (54 °C *vs* 55 °C) and is slightly lower than that of the parent protein ANG^WT^ (59 °C) [18], on which it is useful to underline that the C-terminal 6xHis tag is absent. These data indicated that the applied substitutions (R31Q/R32Q) do not influence the overall thermal stability of the protein. A similar trend was also obtained by qualitatively evaluating the catalytic properties of R31Q/R32Q, (Fig. 2 panel D), using as substrate yeast tRNAs. In particular, we observed that the activity of R31Q/R32Q is practically equivalent to that of ANG.

### 3.2 Subcellular localization of R31Q/R32Q in HaCaT cells subjected to different growth conditions

Prior to verify whether R31Q/R32Q was actively internalized in HaCaT cells and to investigate its subcellular localization, it was necessary to analyse if this mutant protein preserves ANG typical biocompatibility. In this regard, we first performed MTT assays by incubating HaCaT cells for 24 h in the presence of increasing amounts of R31Q/R32Q. The obtained data reported in Fig. S1 (panel A) did not highlight significant toxic effects of R31Q/R32Q, not even at its maximum dose (20 μM). Subsequently, an investigation by immunofluorescence was also carried out, in the conditions selected for the following experiments, to verify whether any cellular morphological alterations or the presence of SGs occur upon treatment with R31Q/R32Q variant. As shown in Fig. S1 - panel B, by incubating the cells for 60 min in the presence of 2 μM R31Q/R32Q, no significant alteration of cell morphology was observed as well as no appearance of SGs was detected by following the distribution of a well-known SGs marker as PABP (Poly-Adenine Binding Protein). Overall, in the experimental conditions used, both tests allowed us to assert that R31Q/R32Q did not present significant toxicity, in an entirely similar way to what has already been verified and described for the parental protein ANG and ANG^WT^ [17].

**Fig. 3:**
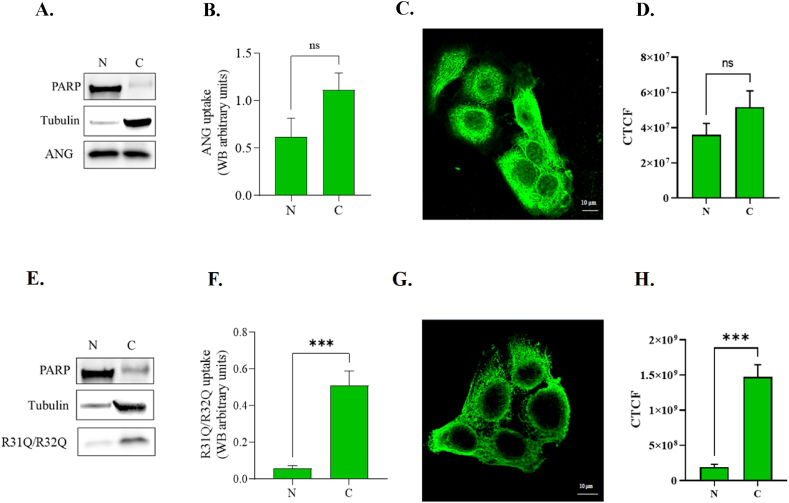
Internalization of R31Q/R32Q and ANG into HaCaT cells. Panels A, B, E and F: western-blotting analysis and quantification on nuclear (N) or cytoplasmic (C) extracts from HaCaT cells treated for 1 h with 2 μM R31Q/R32Q or 2 μM ANG; Tubulin was used as cytosolic marker; poly (ADP-Ribose) polymerase (PARP) was used as nuclear marker. Panels C, D, G and H: immunofluorescence internalization analysis and CTCF (corrected total cell fluorescence) of HaCaT cells treated for 1 h with 2 μM R31Q/R32Q or 2 μM ANG. Green signal was specific for the 6xHis-Tag. (For interpretation of the references to color in this figure legend, the reader is referred to the Web version of this article.)

Once verified the absence of cytotoxicity of R31Q/R32Q, western-blotting and immunofluorescence analyses were carried out to investigate protein internalization into HaCaT cells and its subcellular localization. On this regard, in Fig. 3 it was possible to ascertain that R31Q/R32Q is effectively internalized in selected cells and, even more interestingly, its subcellular localization was found to be predominantly cytosolic. Indeed, in Fig. 3 (panels E–F) the electrophoretic analyses performed on nuclear and cytoplasmic protein extracts indicated that R31Q/R32Q preferentially accumulates in the cytoplasm, and this is definitely in contrast with what it was observed for the parental protein which distributes equally between the nucleus and the cytoplasm (Fig. 3 panels A–B). Immunofluorescence images (Fig. 3 panel C for ANG and panel G for R31Q/R32Q) as well as fluorescence intensity analyses (Fig. 3 panel D for ANG and panel H for R31Q/R32Q) also confirmed this trend, thus highlighting a clear topological difference between the two proteins, and validating that the substitutions adopted in the variant R31Q/R32Q really alter its ability to translocate to the nucleus once internalized.

### 3.3 Stress response properties of R31Q/R32Q

**Fig. 4:**
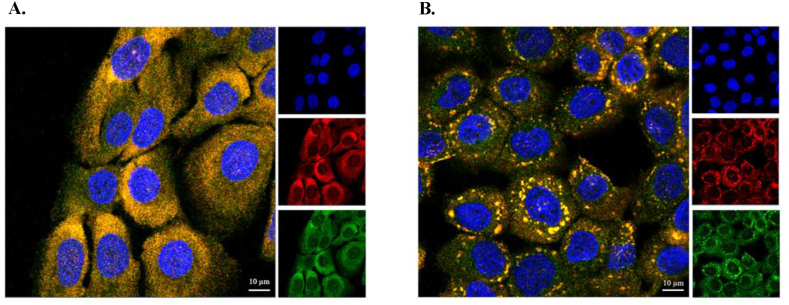
R31Q/R32Q subcellular localization under stress conditions. A. HaCaT cells treated with 2 μM R31Q/R32Q for 1 h; B. HaCaT cells treated with 2 μM R31Q/R32Q for 1 h and then subjected to oxidative stress by SA (500 μM for 1 h). Colour code: blue, nuclei stain by 4′,6-diamidino-2-phenylindole (DAPI); red, Poly(A)-binding protein (PABP); green, R31Q/R32Q; yellow, merge. See Methods section for experimental details. (For interpretation of the references to color in this figure legend, the reader is referred to the Web version of this article.)

The above indications on R31Q/R32Q prompted us to verify whether its subcellular localization was influenced by an alteration of cell homeostasis. On this regard, an immunofluorescence experiment was set up in which HaCaT cells were firstly incubated with R31Q/R32Q (Fig. 4 panel A) and then subjected to the action of 500 μM SA for 1 h. As shown in Fig. 4 (panel B), following this ailment, R31Q/R32Q exhibited dynamic re-localization effectively gathering at the level of cytosolic aggregates attributable to SGs. This behaviour, fully in line with what concerns the unmodified protein [17], clearly suggests that the introduced mutations force the variant protein to localize in the cytosol without however affecting its recruitment on the granules during the stress response. However, the main objective of this work was to verify whether a purely cytosolic localization conferred to R31Q/R32Q a noteworthy contribution to the recovery operations that the cell implements in response to stress. About that, we proceeded to perform a series of tests to accurately probe whether R31Q/R32Q had properties distinguishable from the parental protein in stress recovery operations.

**Fig. 5:**
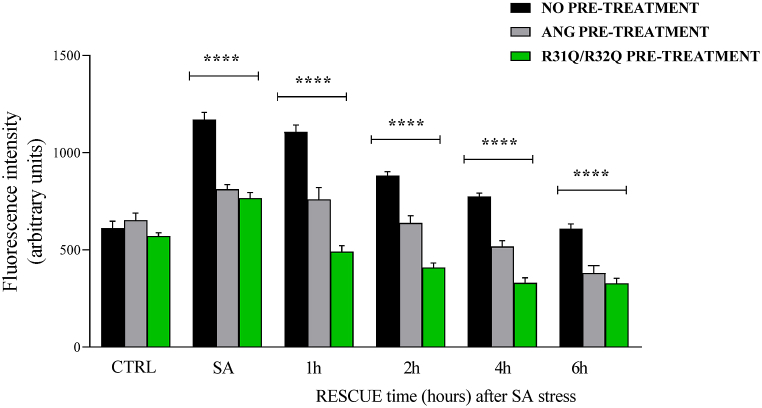
Stress response and rescue analysis in HaCaT cells *via* intracellular ROS detection by DCFH-Da assay. Colour code: black, HaCaT cells not treated beforehand; grey, HaCaT cells pre-treated with 2 μM ANG; green, HaCaT cells pre-treated with 2 μM R31Q/R32Q. CTRL: unstressed cells; SA: cells subjected to sodium arsenite treatment (500 μM for 1 h). Values are the means ± SEM of biological replicates (*p < 0.05, **p < 0.01, ***p < 0.001, ****p < 0.0001) compared to the respective controls (one-way ANOVA, followed by Bonferroni's post-test). See Methods section for experimental details. (For interpretation of the references to color in this figure legend, the reader is referred to the Web version of this article.)

In the previous report [17], a significant contribution by recombinant ANG was ascertained on the attenuation of accumulated ROS in HaCaT cells subjected to stress induced by SA. On the wave of this indication, we set up a ROS quantification analysis in stressed HaCaT cells in which the possible contribution of R31Q/R32Q was compared to that of ANG at 4 different times following stress (1, 2, 4 and 6 h). As shown in Fig. 5, at least two important indications from this test have emerged, namely a significantly lower accumulation of ROS in HaCaT cells pretreated with R31Q/R32Q with respect to those pretreated with unmodified ANG, and a faster recovery of basal ROS with respect to the same process observed in ANG-pretreated cells. In fact, in Fig. 5, HaCaT cells pre-incubated with R31Q/R32Q showed a level of ROS comparable to that of control cells already after 1 h, while in the case of non-pretreated cells and of cells pretreated with ANG, the full recovery of the basal level of ROS was completed in a longer time. In Supplementary Fig. S2 is possible to observe the same data grouped according to the pre-treatment of the cells. The contribution of R31Q/R32Q to limiting ROS release is evident also in this alternative graphics mode.

**Fig. 6:**
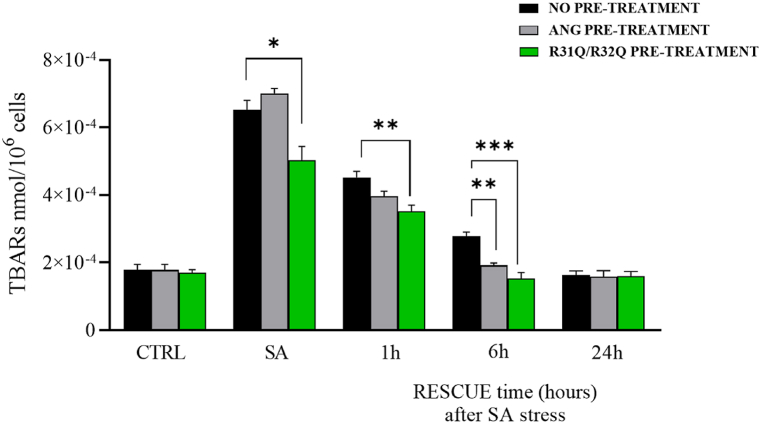
Stress response and rescue analysis in HaCaT cells by evaluation of lipid peroxidation (TBARS assay). Colour code: black, HaCaT cells not treated beforehand; grey, HaCaT cells pre-treated with 2 μM ANG; green, HaCaT cells pre-treated with 2 μM R31Q/R32Q. CTRL: unstressed cells; SA: cells subjected to sodium arsenite treatment (500 μM for 1 h). Values are the means ± SEM of biological replicates (*p < 0.05, **p < 0.01, ***p < 0.001) compared to the respective controls (one-way ANOVA, followed by Bonferroni's post-test). See Methods section for experimental details. (For interpretation of the references to color in this figure legend, the reader is referred to the Web version of this article.)

To better investigate the involvement of R31Q/R32Q in the amelioration of oxidative stress, we also used an alternative method to quantify intracellular levels of ROS, such as the evaluation of lipid peroxidation. For this reason, following the same experimental conditions above described but layered over three different times downstream the oxidative perturbation (1, 6 and 24 h), we analyzed the contribution of R31Q/R32Q to stress response by TBARS assay, a procedure that allows to obtain an indirect measure of lipid peroxidation in stressed cells (see Methods). Interestingly, as indicated in Fig. 6, also by this test it appeared that HaCaT cells preventively pre-treated with R31Q/R32Q and then subjected to SA stress presented an amelioration trend faster than that relative to HaCaT cells pre-incubated with ANG. In detail, in Fig. 6 it was observed that HaCaT cells pre-treated with R31Q/R32Q responded to the oxidative stimulus with a lower degree of lipid peroxidation if compared to untreated cells or cells pre-incubated with ANG. It is also worth noting that basal lipid peroxidation levels in HaCaT cells, both those pre-incubated with R31Q/R32Q and those pre-incubated with ANG, were restored already after 6 h from the oxidizing stimulus, thus highlighting that both proteins have a non-negligible cellular protective effect. In Supplementary Fig. S3 is possible to observe the same data but grouped according to the pre-treatment of the cells. The contribution of R31Q/R32Q to limiting lipid peroxidation is evident also in this alternative graphics mode.

**Fig. 7:**
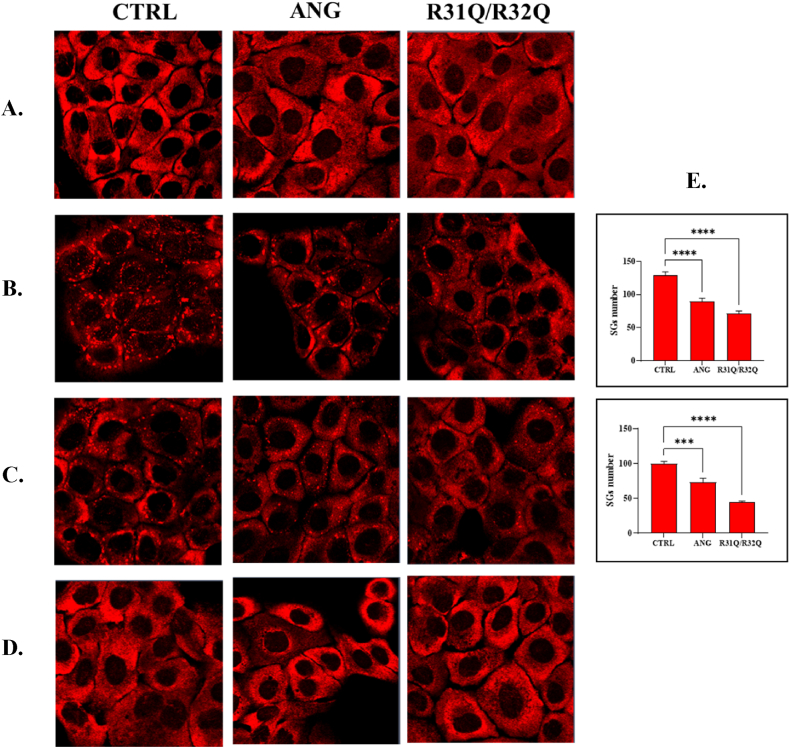
SGs assembly/disassembly analysis by immunofluorescence. A. Unstressed HaCaT cells; B. HaCaT cells subjected to oxidative stress by 500 μM SA for 1 h; C. Recovering HaCaT cells after 1 h; D. Recovering HaCaT cells after 6 h. E. SGs counting relative to panels B and C. Legend: CTRL: untreated HaCaT cells; R31Q/R32Q: HaCaT cells pre-treated with 2 μM R31Q/R32Q for 1 h; ANG: HaCaT cells pre-treated with 2 μM ANG for 1 h. PABP: marker of SGs. Quantification of SGs was obtaining by their count in at least 8 different 63x confocal images and reported as mean ± SEM. Analysis of variance has been performed by One-way ANOVA followed by Bonferroni post-test. (***p < 0.001, ****p < 0.0001) compared with SA. See Methods section for experimental details.

Since even the number of SGs in formation or those gradually remaining downstream of a stressful stimulus can give an insight into the actual rate of cell recovery, we evaluated by immunofluorescence analysis their amount in stressed or recovering HaCaT cells using PABP as biomarker. As indicated in Fig. 7, PABP was widespread and predominantly cytosolic in unstressed cells (panel A), both those untreated and those pre-treated with ANG or R31Q/R32Q, while it colocalized with SGs when cells were treated with SA (panel B). However, what is interesting to note is that both in stressed and in recovering HaCaT cells preliminary treated with R31Q/R32Q, a reduced number of SGs was detected with respect to that found in HaCaT cells pre-treated with ANG (panel C), while it should be observe that after 6 h PABP distribution is completely recovered as the control (panel D). If a more limited number of SGs can be associated with a faster recovery of cell translational activity, this result adds up to indications previously collected in terms of ROS and lipid peroxidation detection and would lead us to suppose that the variant R31Q/R32Q, once internalized, substantially supports the cells in their reaction to stress stimuli.

**Fig. 8:**
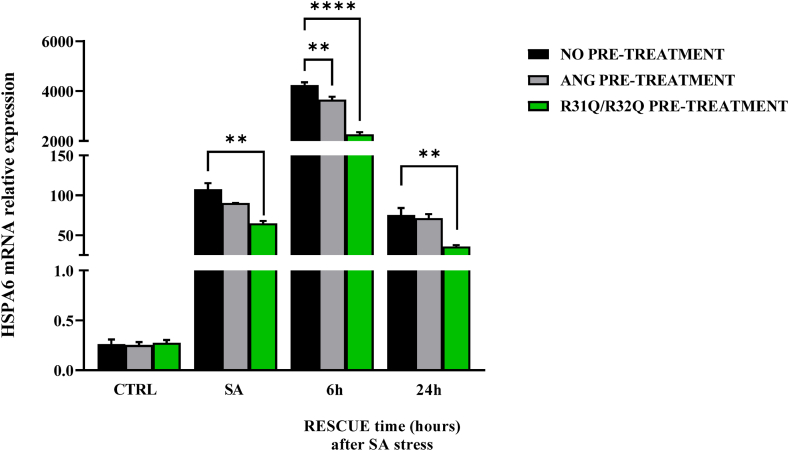
RT-qPCR analysis of the expression of HSPA6 gene. Colour code: black, HaCaT cells not treated beforehand; grey, HaCaT cells pre-treated with 2 μM ANG; green, HaCaT cells pre-treated with 2 μM R31Q/R32Q. CTRL: unstressed cells; SA: cells subjected to sodium arsenite treatment (500 μM for 1 h). Values are the means ± SEM of biological replicates (**p < 0.01, ****p < 0.0001) compared to the respective controls (one-way ANOVA, followed by Bonferroni's post-test). See Methods section for experimental details. (For interpretation of the references to color in this figure legend, the reader is referred to the Web version of this article.)

However, to obtain undoubted evidence of the abilities of this angiogenin variant, we decided to verify whether its action also concretely influences the gene expression of specific cellular markers. Starting from what was previously observed for ANG [17], the first approach consisted in testing the possible contribution of R31Q/R32Q in the attenuation of Heat Shock 70 kDa Protein 6 (HSPA6) expression in HaCaT cells stimulated with SA. It is useful to underline that the significance of HSPA6 expression in epidermal keratinocytes, both for its chaperoning contribution as well as for stress-protective functions, is well documented [43]. As reported in Fig. 8, the expression level of HSPA6, naturally increasing during SA administration and even more evident in the first 6 h of recovery, was significantly attenuated in HaCaT cells previously treated with R31Q/R32Q. It is noteworthy that the detected expression reduction was found to be more pronounced than that observed for the untreated cells and for the cells treated with ANG, thus highlighting once again that this variant appears to have more marked cellular rescue properties than the parental protein.

**Fig. 9:**
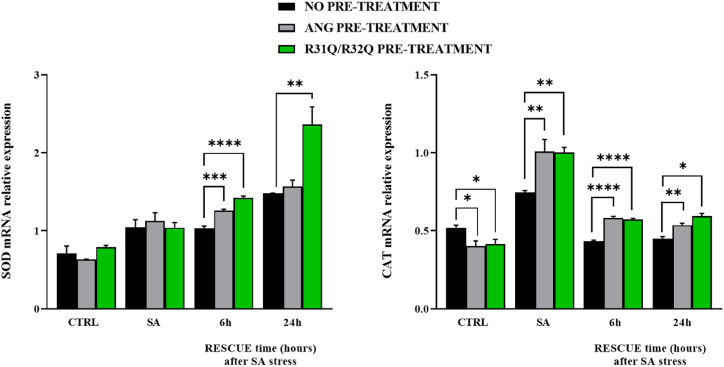
RT-qPCR analysis of the expression of SOD and CAT genes. Colour code: black, HaCaT cells not treated beforehand; grey, HaCaT cells pre-treated with 2 μM ANG; green, HaCaT cells pre-treated with 2 μM R31Q/R32Q. CTRL: unstressed cells; SA: cells subjected to sodium arsenite treatment (500 μM for 1 h). Values are the means ± SEM of biological replicates (**p < 0.01, ****p < 0.0001) compared to the respective controls (one-way ANOVA, followed by Bonferroni's post-test). See Methods section for experimental details. (For interpretation of the references to color in this figure legend, the reader is referred to the Web version of this article.)

However, to further validate the pronounced protective potential of R31Q/R32Q, we investigated by RT-PCR its influence on the expression of other key cellular markers involved in the protection from radical attack. In a first approach we chose a gene pair encoding enzymes directly implicated in the conversion of ROS, as catalase (CAT) and superoxide dismutase (SOD). As reported in Fig. 9, the expression profile of SOD in stressed cells increased during their recovery, clearly indicating that this metalloenzyme is strictly necessary for SA oxidative response as indicated by its scavenger role of destroying free superoxide radicals; interestingly, in HaCaT cells pre-treated with R31Q/R32Q, SOD expression was particularly accentuated in a time-dependent manner. When the effect of R31Q/R32Q on the other selected gene (CAT) was observed, instead, a contribution similar to that of the parental protein was observed either during the stress, when the expression of CAT was essential, or during an initial phase of recovery of the cells (6 h) during which the expression of CAT also began to decrease. However, a significant difference between the contributions of R31Q/R32Q and unmodified ANG was detectable at longer times, thereby highlighting that R31Q/R32Q was able to perpetuate its contribution to the cellular response (see Fig. 9).

**Fig. 10:**
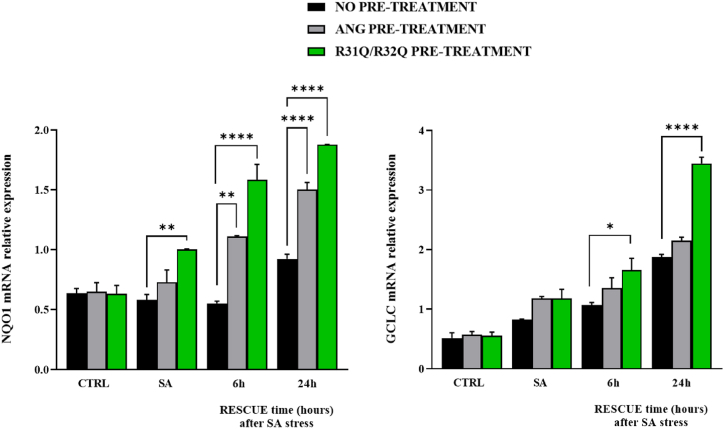
RT-qPCR analysis of the expression of NQO1 and GCLC genes. Colour code: black, HaCaT cells not treated beforehand; grey, HaCaT cells pre-treated with 2 μM ANG; green, HaCaT cells pre-treated with 2 μM R31Q/R32Q. CTRL: unstressed cells; SA: cells subjected to sodium arsenite treatment (500 μM for 1 h). Values are the means ± SEM of biological replicates (**p < 0.01, ****p < 0.0001) compared to the respective controls (one-way ANOVA, followed by Bonferroni's post-test). See Methods section for experimental details. (For interpretation of the references to color in this figure legend, the reader is referred to the Web version of this article.)

Since the ability of angiogenin to activate the Nrf2/ARE pathway and thus to contribute significantly to the maintenance of redox homeostasis has been established in some cell lines [44], we tried to obtain a further validation of the abilities of R31Q/R32Q also on stressed HaCaT cells. Based on these considerations, in a second approach we selected two genes both regulated by Nrf2/ARE pathway: NQO1 (NAD(P)H quinone dehydrogenase 1) and GCLC (Glutamate-cysteine ligase catalytic subunit). As reported in Fig. 10, again we collected indications on R31Q/R32Q similar to those above reported on its propensities to accentuate the expression of specified genes. In detail, we observed that the expression of NQO1 and GCLC genes in stressed R31Q/R32Q pre-treated HaCaT cells was always more pronounced, in a time-dependent manner, with respect to that detected in stressed untreated or ANG pre-treated HaCaT cells. In our opinion, this result arouses particular interest for two reasons: the former, because it confirms the potential of R31Q/R32Q with respect to the parental protein and the latter, because the antioxidant contribution *via* Nrf2/ARE pathway by angiogenin had never been documented before in HaCaT cells.

## 4 Discussion

Human ANG is undoubtedly the most versatile vertebrate secreted RNase both for the number of its specific interactors identified to date [[45], [46], [47], [48]] and for the cellular functions in which it is involved [49]. One of the most recently discovered properties attributed to ANG is related to its stress-induced ability to hydrolyse tRNAs [30,50] and mediate the restoration of a new cellular homeostasis [18,51,52]. The decisive aspect regarding these stress-induced activities of ANG is represented by its subcellular localization which must necessarily be cytoplasmic. This, in addition to dissociation from its inhibitor (RNHI), clearly implies a consistent dynamism both for nuclear ANG [15], which must perform the necessary retro-translocation towards the cytoplasm, and for ANG taken up from the extracellular environment that must converge on the appropriate sites to hydrolyse tRNAs and assist in the SGs recruitment process [33,53,54]. Precisely in relation to this last statement, we engineered a recombinant variant of ANG (R31Q/R32Q) in which the NLS region has been profoundly altered (Fig. 1) to substantially minimize its possible translocation in the nucleus once internalized and test its stress-induced attitudes compared with the unmodified protein. This approach takes its cue from the need to start laying concrete foundations for understanding the stress-induced mechanism of ANG. In addition to this, this strategy was conceived on the wave of encouraging results obtained in our previous report in which we highlighted an important contribution mediated by recombinant ANG in the response to stress of human keratinocytes. Based on these assumptions, we analyzed R31Q/R32Q for its conformational and catalytic properties (Fig. 2) and, while not detecting significant differences with respect to parental protein, we verified that it was effectively internalized into keratinocytes and that its localization complied with the purposes induced by substitutions introduced in NLS region. With these premises, we have gradually deepened the effects of R31Q/R32Q and we demonstrated that, in addition to not having undesirable effects in terms of vitality or alterations of cell homeostasis (Fig. S1), this variant concentrated mainly in the cytosol (Fig. 3) and, quite crucial for the aims of this manuscript, on SGs when cells were subjected to oxidative stress (Fig. 4). Ascertained this, we carefully probed its subcellular localization and contribution to stress response in close comparison with the recombinant parental protein of which some stress-induced abilities have been reported previously [17]. The first goal we set out was to test the possible contribution of R31Q/R32Q to the mitigation of the accumulation of ROS (Fig. 5 and Fig. S2) and lipid peroxidation. (Fig. 6 and Fig. S3). The choice of these two parameters has allowed us to obtain an immediate picture of the degree of stress response of HaCaT cells mediated by R31Q/R32Q. By this way, we highlighted that this variant contributed substantially to cell defence and always more effectively than the parental protein. This, in addition to the consideration that both proteins share the same folding and the same catalytic properties, is the first real indication that perhaps a higher cytosolic density could support beneficial effects during the restoration of physiological functions after a perturbation. Further supporting data were also obtained by analysing the number of SGs detected in HaCaT cells previously treated with R31Q/R32Q or ANG and then subjected to treatment with SA. Again, we observed a clear difference between the effects of R31Q/R32Q and ANG (Fig. 7). Indeed, the lower number of SGs associated with the treatment with R31Q/R32Q supports the idea that the pre-treatment with the variant in HaCaT cells has tangible effects on the recovery from the stress. Indeed, it should be remarked that SGs are aggregations of translationally silent ribonucleoproteins in which the cell uses to concentrate its anabolic resources on the active counter-offensive to stress and to restore a new homeostasis. If the restoration of homeostasis requires the assembly of fewer SGs, then we could hypothesize that concurrently the cells implement the expression of specific genes and, to comply with this strategy, they also draw on components of SGs, including ANG. In relation to this suggestive picture, it seemed reasonable to test the ability of R31Q/R32Q to influence the gene expression of some molecules directly involved in the stress response and the indications obtained in our opinion are rather interesting. In the first instance we found that R31Q/R32Q was quite adept at attenuating the expression of heat shock protein HSPA6 in stressed HaCaT cells (Fig. 8) and this is noteworthy since the association between this effect and the reduction of SGs induced by the same protein (Fig. 7) allows for an interesting reflection. Indeed, by observing Fig. 8, R31Q/R32Q appears able to moderate the expression of HSPA6 both in the moments immediately following the oxidizing action of SA and during the cell recovery following the exposure to the stress stimulus. This, in addition to indicating a lasting effect of R31Q/R32Q, leads to imagine an interaction of the protein with specific partners to affect the transcription pathway of HSPA6. To support this hypothesis, we have collected additional achievements, and all would confirm a certain susceptibility of other stress-induced genes as SOD and CAT (Fig. 9) or NQO1 and GCLC (Fig. 10) to R31Q/R32Q. Indeed, SOD and CAT encode enzymes directly involved in the attenuation of ROS accumulation in stressed cells, whereas NQO1 and GCLC are involved in antioxidant Nrf2/ARE pathway. For all the considered genes, we found the same trend, namely that HaCaT cells pre-treated with R31Q/R32Q, once perturbed, are characterized by a stimulation of the expression of the genes under test higher than that observed for untreated cells and, in most of the cases, than that observed for ANG pre-treated cells.

## 5 Conclusions

The above indications would therefore suggest an active predisposition of this variant to alter the expression of several genes during the stress response. If we consider that these aptitudes have been enhanced by compromising the ability of the protein to translocate to the nucleus, it is reasonable to assume that angiogenin, in addition to its direct contribution to release tiRNAs, is inherently eligible to be involved in the cytosol in a molecular network capable of managing the stress response also at the gene level. This allows to hypothesize that the effects of angiogenin could be of even more marked relevance considering that its activity is strictly related to its availability and, in this regard, skin cells or any other cell type suitable for a direct therapeutic administration could be excellent candidates to explore the therapeutic and/or protective potentialities of this special RNase.

## Data availability

Data will be made available on request.

## CRediT authorship contribution statement

**Rosanna Culurciello:** Writing – original draft, Validation, Methodology, Conceptualization. **Ilaria Di Nardo:** Methodology, Investigation. **Andrea Bosso:** Methodology, Investigation. **Francesca Tortora:** Methodology, Investigation. **Romualdo Troisi:** Methodology, Investigation. **Filomena Sica:** Writing – review & editing, Supervision. **Angela Arciello:** Writing – review & editing, Formal analysis. **Eugenio Notomista:** Validation, Supervision. **Elio Pizzo:** Writing – review & editing, Writing – original draft, Supervision, Funding acquisition, Conceptualization.

## Declaration of competing interest

The authors declare that they have no known competing financial interests or personal relationships that could have appeared to influence the work reported in this paper.
