# Supplementary figures and images for: Tailoring the stress response of human skin cells by substantially limiting the nuclear localization of angiogenin
Source: Heliyon. 2024 Jan 21;10(3):e24556. doi: 10.1016/j.heliyon.2024.e24556 (PMC10839879; doi:10.1016/j.heliyon.2024.e24556)

## Slide 1
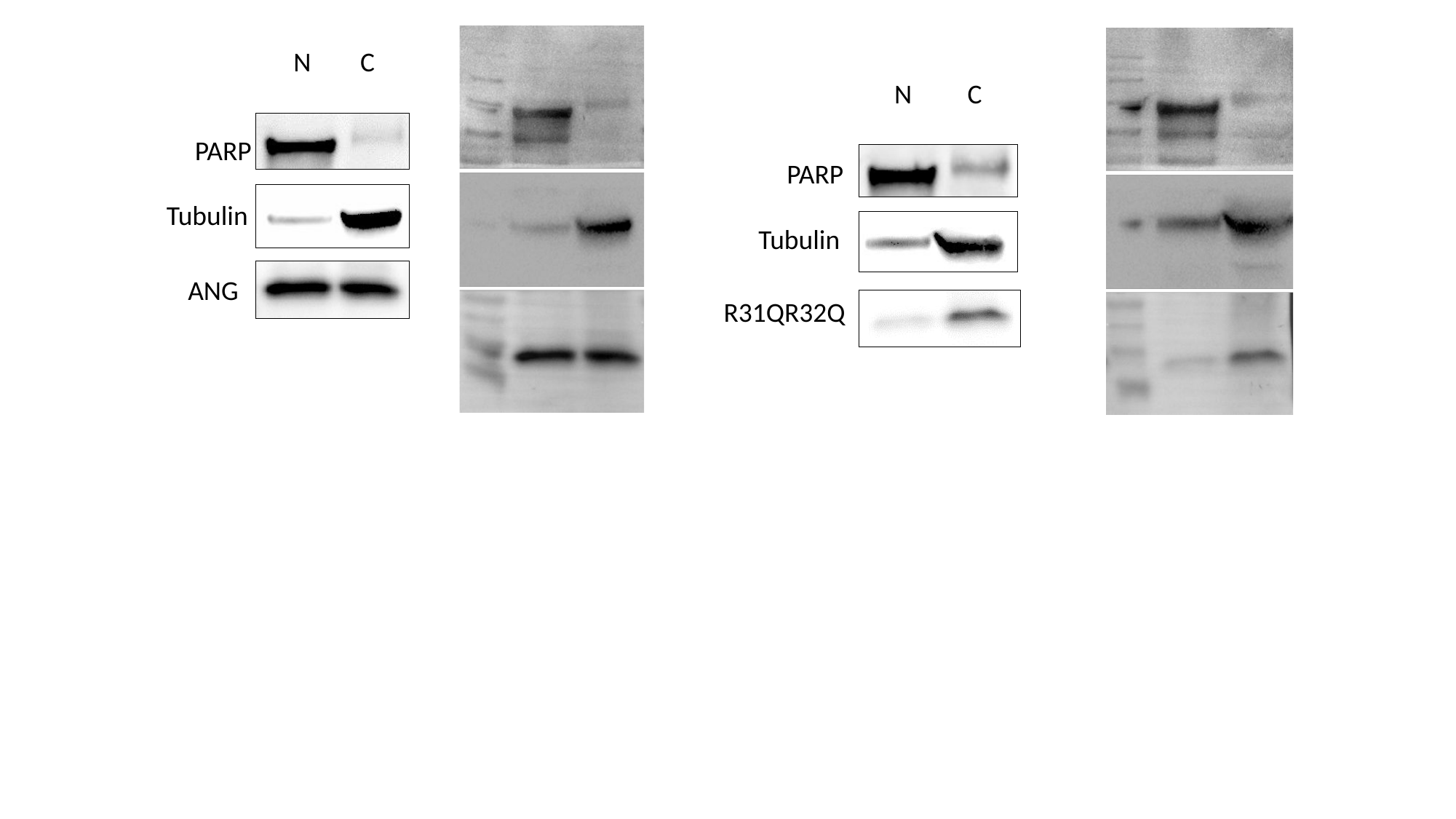

N C
N C
PARP
PARP
Tubulin
Tubulin
ANG
R31QR32Q

Supplement: Multimedia component 1 [file mmc1.pptx]
