# Supplementary material for: Tailoring the stress response of human skin cells by substantially limiting the nuclear localization of angiogenin
Source: Heliyon. 2024 Jan 21;10(3):e24556. doi: 10.1016/j.heliyon.2024.e24556 (PMC10839879; doi:10.1016/j.heliyon.2024.e24556)
Supplement: Multimedia component 2 [file mmc2.docx]

**Tailoring the stress response of human skin cells by substantially limiting the nuclear localization of angiogenin**

Rosanna Culurciello^1^, Ilaria Di Nardo^1^, Andrea Bosso^1^, Francesca Tortora^1^, Romualdo Troisi^2,3^, Filomena Sica^2^, Angela Arciello^2^, Eugenio Notomista^1^ and Elio Pizzo ^1,4*^

^1^ Department of Biology, University of Naples Federico II, 80126 Naples, Italy;

^2^ Department of Chemical Sciences, University of Naples Federico II, 80126 Naples, Italy;

^3^ Institute of Biostructures and Bioimaging, CNR, 80131 Naples, Italy;

^4^ Centro Servizi Metrologici e Tecnologici Avanzati (CeSMA), University of Naples Federico II, 80126 Naples, Italy

*** Corresponding author**

Elio Pizzo: elipizzo@unina.it Tel. +39-081679151


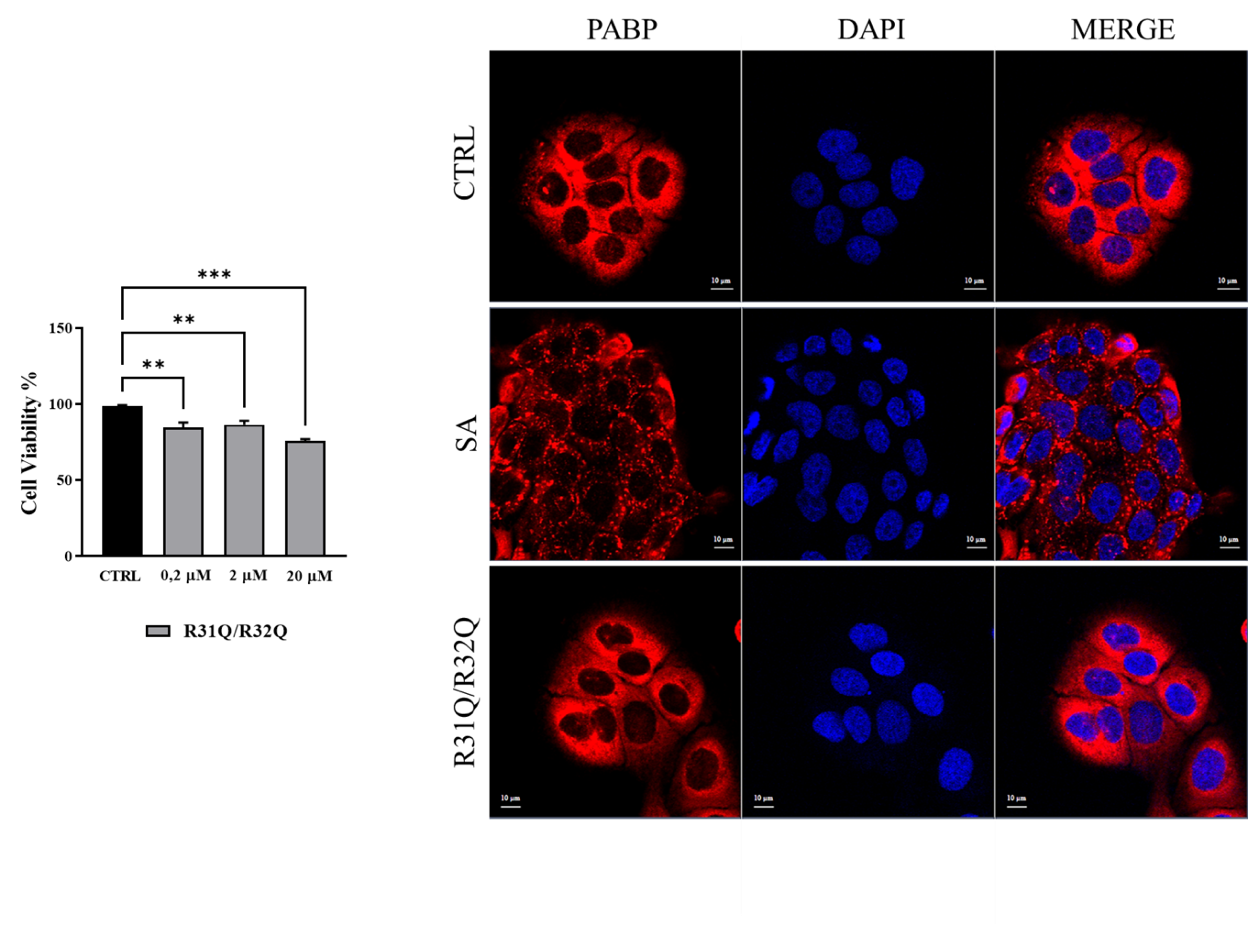


**Figure S1: Analysis of biocompatibility of R31Q/R32Q in HaCaT cells. A. MTT assays with increasing doses of R31Q/R32Q on HaCaT cells after 24 h treatment. B. Immunofluorescence analysis of HaCaT cells treated with 2 µM of R31Q/R32Q for 1 h. The administration of 500 µM of SA for 1 h was used as positive control of SGs formation. Colour code: red, Poly(A)-binding protein (PABP); blue, nuclei stain by 4′,6-diamidino-2-phenylindole (DAPI). See Methods section for experimental details.**

**C.**

**B.**

**A.**


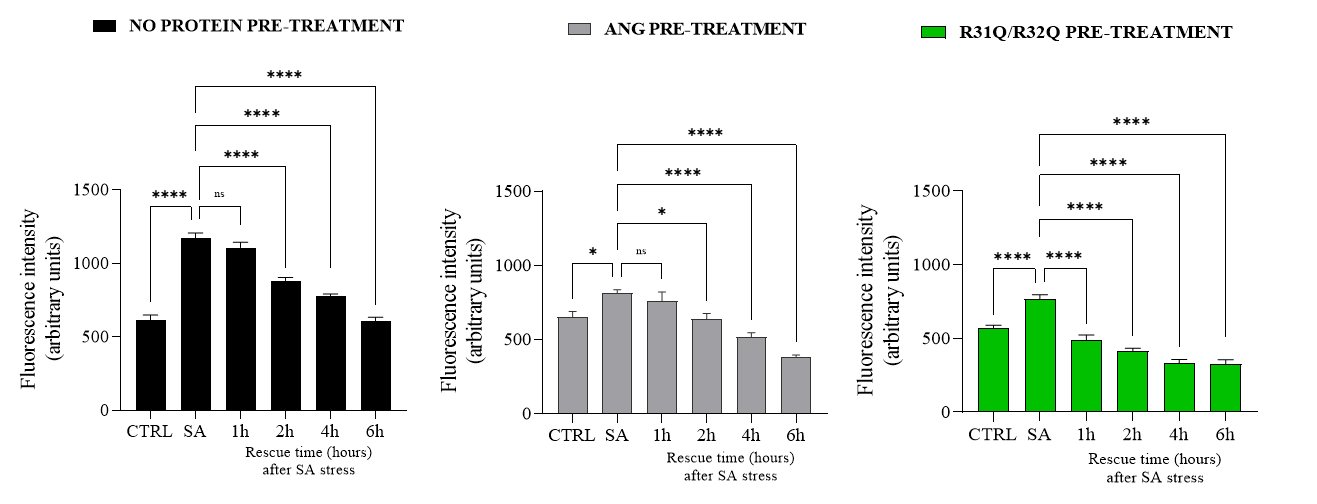


**Figure S2. Stress response and rescue analysis in HaCaT cells via intracellular ROS detection by DCFH-Da assay. A. HaCaT cells not treated beforehand; B. HaCaT cells pre-treated with 2 µM ANG; C. HaCaT cells pre-treated with 2 µM R31Q/R32Q. CTRL: unstressed cells; SA: cells subjected to sodium arsenite treatment (500 µM for 1 h). Values are the means ± SEM of biological replicates (* p < 0.05, ** p < 0.01, *** p < 0.001) compared to the respective controls (one-way ANOVA, followed by Bonferroni’s post-test). See Methods section for experimental details.**


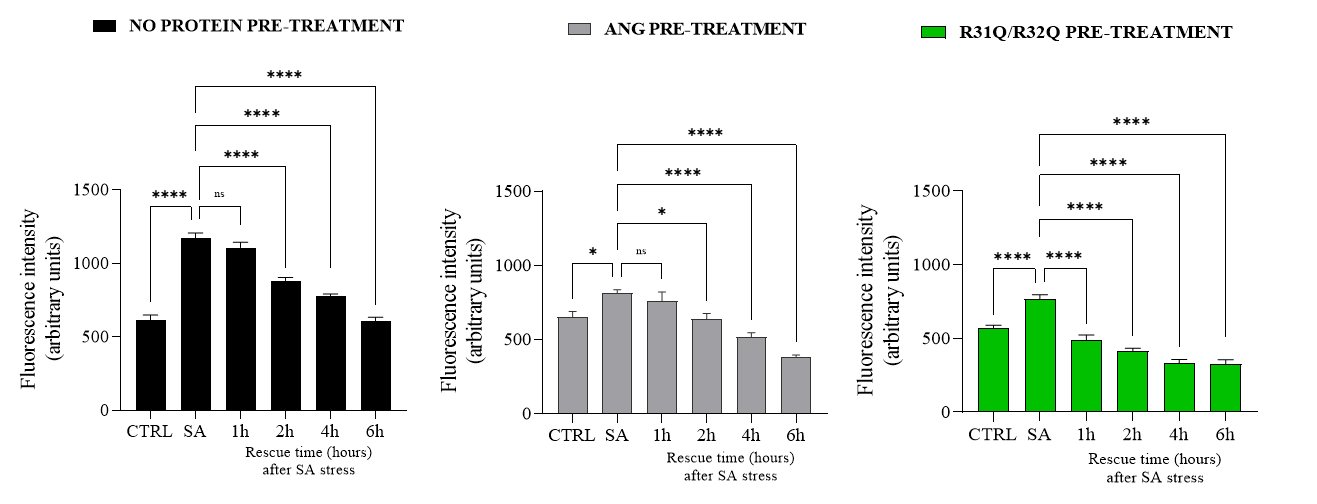


**Figure S3. Stress response and rescue analysis in HaCaT cells by evaluation of lipid peroxidation (TBARS assay). A. HaCaT cells not treated beforehand; B. HaCaT cells pre-treated with 2 µM ANG; C. HaCaT cells pre-treated with 2 µM R31Q/R32Q. CTRL: unstressed cells; SA: cells subjected to sodium arsenite treatment (500 µM for 1 h). Values are the means ± SEM of biological replicates (* p < 0.05, ** p < 0.01, *** p < 0.001) compared to the respective controls (one-way ANOVA, followed by Bonferroni’s post-test). See Methods section for experimental details.**
